# Relationship of self-reported pica and avoidant restrictive food intake disorder symptomology with dimensions of impulsivity, perceived stress among Pakistani University students

**DOI:** 10.1186/s40337-023-00956-z

**Published:** 2024-01-22

**Authors:** Sara Aleem Haqqi, Siddrah Irfan

**Affiliations:** National University of Sciences and Technology, Islamabad, Pakistan

**Keywords:** Pica, Avoidant restrictive food intake disorder, Dimensions of impulsivity, Stress, Young adults

## Abstract

**Introduction:**

Pica and avoidant/restrictive food intake disorder are two of the three new eating and feeding disorders introduced in the DSM-5, this inclusion has drawn attention to the immediate need for research into their prevalence, diagnostic assessment, underlying risk factors and optimal treatment. There are very few studies available that explored the predictors or causes of these eating disorders specifically in Pakistani context.

**Objectives:**

The objectives of the current study include (a) to estimate the presence of pica and avoidant/restrictive food intake disorder symptomology among young adults in Pakistan, (b) to examine the relationship between dimensions of impulsivity, stress and presence of pica and avoidant/restrictive food intake disorder, (c) to explore the mediation role of stress.

**Methods:**

The sample consisted of 660 young adults with age range 18–25 years, recruited through convenient sampling. The respondents were provided with a questionnaire comprising of an informed consent, a demographic information sheet and self-report measures including PARDI-AR-Q to assess symptomology of pica and avoidant/restrictive food intake disorder, UPPS-P Impulsive Behaviour Scale to assess dimensions of impulsivity and Perceived Stress Scale to measure perceived stress.

**Results:**

The findings show that 28 participants (4.2%) reported Pica symptomology, of which eleven participants indicated that they currently consume more than one non-food item. 19 respondents (2.8%) reported avoidant/restrictive food intake disorder symptomology. 13 respondents reported varying degrees of all three symptom clusters namely sensory based food avoidance, lack of interest in food, and fear of negative consequences of eating. Additionally, each of the dimensions of impulsivity was found to be positively correlated to the presence of pica and avoidant/restrictive food intake disorder. Perceived stress was identified as a mediating factor between each of the dimensions of impulsivity and presence of pica and avoidant/restrictive food intake disorder.

**Conclusion:**

Although current study focused on a particular age range, it has drawn attention to the necessity of screening and investigating other strata of Pakistani population for pica and avoidant/restrictive food intake disorder. The clinical utility of the present research resided in the identification of factors associated with pica and avoidant/restrictive food intake disorder, an information which can be used to tailor psychological interventions, modify existing ones, and inform the future research on evidence-based treatment.

## Abstract (plain language summary)

The study explored pica and avoidant/restrictive food intake disorder in young adults in Pakistan. It found a presence of these disorders and identified connections with impulsivity and stress. These disorders, characterized by unusual eating behaviors and food avoidance, showed links with impulsivity dimensions and stress levels. Importantly, stress seemed to play a role between impulsivity and these disorders. This study emphasizes the need for broader research across different age groups in Pakistan to understand these disorders better. The study suggests that these eating disorders aren’t confined to specific demographics, supporting the DSM-5 decision to lift age limits. In essence, this research opens doors for understanding and addressing pica and avoidant/restrictive food intake disorder in diverse populations, fostering a more comprehensive approach to mental health in the context of eating behaviors.

## Introduction

Recently, the DSM-5 has refined the diagnosis of eating and feeding disorders, introducing new categories like pica and avoidant/restrictive food intake disorder. Pica involves compulsive consumption of non-food items [1], commonly consumed items include clay, ice, charcoal, wood, and paint chips [1]. The health outcomes of consuming non-food items vary by great degree and can even be life threatening [2]. On the other hand, avoidant/restrictive food intake disorder exhibits phenotypic variability with three distinct symptom clusters: sensory sensitivity with preferences for food texture, appearance, and taste; lack of interest or appetite for food; and restrictive food intake due to fear of negative outcomes like choking [3]. The presence of these symptom clusters varies among individuals, with some displaying only one and others may have combination of two or all three [4]. Avoidant/restrictive food intake disorder also leads to various medical complaints over time including severe malnourishment, gastrointestinal discomfort, organ atrophy, nausea, and immunodeficiency [5]. However, if pica and avoidant/restrictive food intake disorder left un diagnosed and untreated, can lead to significant psychosocial and health issues in later stages of life. Therefore, it is pertinent to explore the occurrence of these symptomology and its correlates. Furthermore, globally, there is a lack of research on pica and avoidant/restrictive food intake disorder, particularly in the context of Pakistan and young adults.

Young adulthood is a critical period for the onset of eating disorders [6]. This age group has the highest risk of developing eating disorders, mild symptoms or subthreshold levels of eating disorders present during young adolescence which may turn into severe symptomology during this period [7]. Furthermore, research on pica and avoidant/restrictive food intake disorder was focused on individuals with specific sociodemographic because it was assumed that these conditions were unique to those groups. For instances, avoidant/restrictive food intake disorder was linked to infancy and early childhood (Katzman et al. 2014) and pica was associated with intellectual impairment, pregnancy, and iron deficiency [8]. Besides, research on eating disorders is predominantly focused on females which has created a gap in knowledge about the presentation of the eating disorder symptoms in males and comparison between genders [9]. Thus, pica and avoidant/restrictive food intake disorder remain underreported across diverse age cohorts, gender identities, and cultural contexts. Therefore, there is a need to conduct more research on the presentation of pica and avoidant/restrictive food intake disorder in various cultures and age groups to improve the differential diagnoses and early detection and the predictors for these problems.

Among numerous factors, personality traits and stress have been found to play a significant role in the development and progression of eating disorders [10]. Some of the most studied personality traits in the eating disorder literature are perfectionism, neuroticism, impulsivity, reward dependence, assertiveness, and compulsivity [11]. Impulsivity is recognized as a transdiagnostic feature common across eating disorders and is associated with severity of symptoms and poor treatment outcomes; therefore, it is often a treatment target of psychological interventions for eating disorders [12]. However, there are few evidence-based treatments for avoidant/restrictive food intake disorder, and none yet published for pica, thus investigating transdiagnostic factors, such as impulsivity is an addition in the literature of eating disorders and impulsivity.

Whiteside and Lynam [13] identified four dimensions of impulsivity: urgency, lack of premeditation, lack of perseverance, and sensation seeking. These dimensions represent distinct pathways to impulsivity and each may contribute differently to psychological disorders. For instances, negative urgency increases the vulnerability towards disordered eating which leads to eating disorders [14]. However, the relationship between pica and avoidant/restrictive food intake disorder with negative urgency is understudied. Furthermore, positive urgency has received less attention in connection to the eating disorders despite that it is linked to other psychological disorders marked by impulsivity such as gambling disorder, alcohol use disorder [15]. Besides, Steward et al. [16] reported that women with eating disorders [including binge eating disorder (BED and anorexia nervosa) had higher levels of both negative and positive urgency compared to women without any eating disorders, however, this relationship needs further exploration in both male and female adults, specifically with reference to pica and avoidant/restrictive food intake disorder.

Another dimension of impulsivity is sensation seeking which is related to eating pathology. Previous research reported that adolescent girls with eating disorders characterized by purging and bingeing, regardless of diagnosis, scored higher on sensation seeking compared to girls without symptoms of any eating disorder, whereas girls with restrictive anorexia nervosa scored lower on sensation seeking compared to group without any eating pathology [17]. A similar trend was noted by Castro-Zamudio and Castro-Barea [18] with a different sample comprising of young adults of both young male and female adult. Therefore, it is assumed that there could be link between different presentations of other eating problems and sensation seeking. Moreover, the past studies reported that there is no significant relationship between lack of premeditation and eating problems (Lavender et al. 2017). Overall, the literature presents mixed evidence, and these link needs further investigation. Since pica and avoidant/restrictive food intake disorder are newly classified and there are no evidence-based treatments available. Therefore, it is crucial to understand the underlying psychological mechanisms, transdiagnostic factors, and potential treatment targets for pica and avoidant/restrictive food intake disorder.

**Fig. 1:**
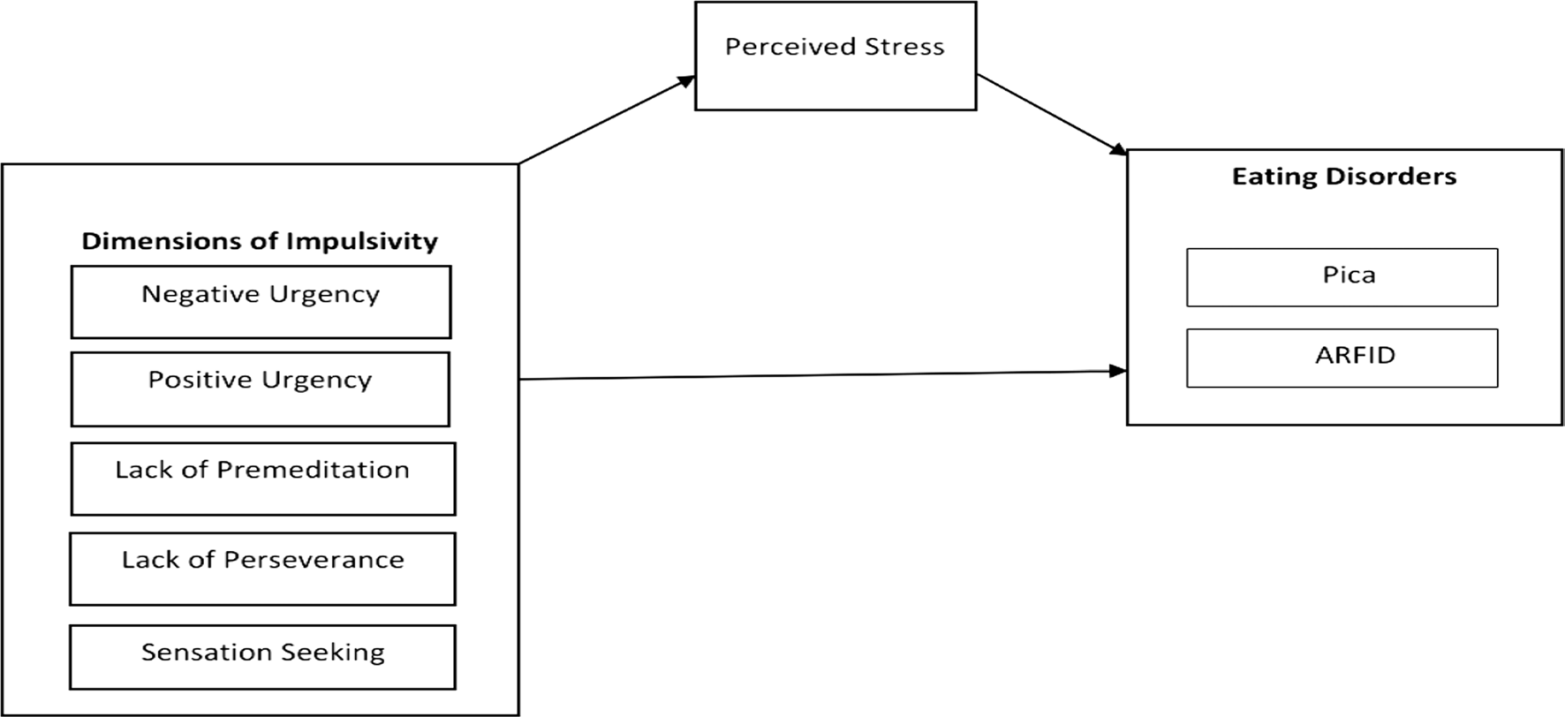
Conceptual framework of the study

Furthermore, diathesis stress model explained the development psychopathology as the interaction between stress and some form of predisposition [19]. Stress is associated with poor health outcomes, mortality, and disease risk [20]. Research suggests that stress affects the health directly by physiological mechanisms but also affects health through indirect processes by modifying health related behaviors such as eating habits [21]. It is reported that during stress individuals make unhealthy food choices over healthy ones and engage in risky eating behaviors in addition to overeating and undereating [22]. Like all other eating disorders, the features mentioned above are also reflected in avoidant/restrictive food intake disorder and pica. However, the direct and indirect effect of stress in these two disorders has not be extensively studied despite that there is evidence for the role of stress in both pica [23] and avoidant/restrictive food intake disorder [24].Therefore, the proposed model for the study determines the unique and interactive effects of personality (diathesis) and perceived stress (environmental variables) on each of the outcome variables (pica and avoidant/restrictive food intake disorder). Figure 1 represents the conceptual framework of the current study.

### Pakistan context

In Pakistan, eating disorders are generally reported by dentists and gastroenterologists [25], and the medical literature is focused on collateral health issues derived from eating disorders instead of the eating disorders that cause those health issues. Pica often goes undiagnosed in Pakistan and most of the research available here is on institutionalized population, in the form of case studies which makes it difficult to estimate the global prevalence rates. The case studies reported pica as a factor associated with pregnancy and helminthic infestations in children from low socioeconomic backgrounds [26, 27, 28]. However, while these studies identify the presence of pica but it is not studied as a primary condition in these studies. Furthermore, these studies are limited to children who live in poor living arrangements. No estimate of frequency is available for the presence of pica in any segment of the Pakistani population. Moreover, in Pakistan unlike pica, there are not any case studies available on avoidant/restrictive food intake disorder nor it is investigated as a factor related to other medical conditions, thus even less is known about its existence. Thus, the literature review highlighted the need to establish the extent of the presence of these disorders, to determine their relevance in Pakistani context and the predictors for these disorders to develop better intervention and prevention plan for the youth of Pakistan facing eating problems. The objectives of the research are as follows:

1. To explore the presence of symptoms of pica and avoidant/restrictive food intake disorder among university students in Pakistan.
2. To determine the relationship between impulsivity with stress and symptomology of pica and avoidant/restrictive food intake disorder among university students in Pakistan.
3. To explore mediating role of stress in relation between dimensions of impulsivity and symptomology of pics and avoidant/restrictive food intake disorder.

## Method

### Procedure

The current study was a cross-sectional survey-based study. The ethical review committee of National University of Science and Technology (NUST), Islamabad granted approval for the current study on September 8, 2021, with IRB No. 09-2021-01/02. Online data collection was approved to ensure that students participating in the study were not at risk of being exposed to COVID-19 because of their participation in the current study. After obtaining the ethical approval a pilot study was conducted after which the online survey was disseminated across the target group through convenience sampling. The survey was posted on Facebook groups of five universities. It was also circulated by IT Admins of the three universities amongst the WhatsApp groups and was shared by members of the target group to their contacts and university groups on social media. Potential participants received detailed information about the study and indicated their consent to participate after arriving at the landing page of the online survey (created on Google Forms). Following that, participants completed the survey. Participants were given an email address to which they could send any questions or concerns. The data was collected from September to November of 2021.

### Sample

The inclusion criterion was young adults aged 18–24 enrolled in Pakistani universities. Due to the COVID-19 situation in Pakistan, convenience sampling technique was used, and data was collected online via google forms. Pica may co-occur with pregnancy and is linked to pregnancy in such a way that when pregnancy ends, pica ends as well [29], so pregnancy was designated as an exclusion criterion. The sample size of this study was determined by using Raosoft and was found to be 660 for confidence interval of 99% and margin of error of 1% [30]. A total of 662 participants (427 females, 221 males, 14 other/prefer not to specify) responded to the survey, of which two were excluded for meeting the exclusion criteria (i.e., pregnancy). The responses were received from 37 cities of Pakistan from Karachi to Gilgit, and the sample included respondents from 15 ethnic groups of Pakistan. Mean age of the participants was 20.85 (SD =  ± 3.53).

### Measures

The protocol consisted of a demographic information sheet and Pica and avoidant/restrictive food intake disorder symptoms, impulsivity Short Urgency, Premeditation (lack of), Perseverance (lack of), and Sensation seeking (UPPS) Impulsive Behavior Scale, Perceived Stress Scale. These measures are briefly described as follows:

#### Demographic Information

The demographic information was included age, gender, marital status, father's education and occupation, mother's education and occupation, number of family members, residential status, income range, ethnic background, total monthly income, employment status, year at university, and subjectively rated academic performance and English proficiency. A question was added on whether the participants had any diagnosed mental health condition. avoidant/restrictive food intake disorder cannot be diagnosed if the food restriction is due to scarcity of food, so an item on unavailability of food also included.

#### Pica and avoidant/restrictive food intake disorder symptoms

PARDI-AR-Q Self 14+ is a self-report avoidant/restrictive food intake disorder measure for individuals over the age of 14 that is based on the Pica, avoidant/restrictive food intake disorder, and Rumination Disorder Interview (PARDI) [31]. It is a 32-item self-report measure which has demonstrated good reliability and validity [31]. It has three versions, the self-report version for adults was used in this research. It was modified with the permission of the author for the current study. One item was added to assess the presence of body weight, shape, or size concern to rule out the presence of anorexia nervosa or any other eating disorder. In addition to that, three more items were added to access relevant supporting information. These added items were not scored but provide the extra information about the symptoms. The first item gauged whether pica behaviour was part of a cultural, religious, or socially normative practise. It was added because DSM-5 suggests that the understanding the symptomology of pica cannot be considered if that eating behaviour is due to any cultural and socially normative practice then the diagnosis should not be assigned [32]. The second item assessed which area of life of the participant is impacted by pica behaviour (mental health, physical health, social life, none). The participants could select multiple options and this item provided an insight into the perceived impact of pica on the life of affected individuals. The third item asked participants if they wanted to seek help for their pica behaviour, those who responded with yes were emailed a list of paid and free of cost services where they could get detailed assessment and proper psychological treatment. The items of PARDI-AR-Q were scored by following the approach adopted by the authors in the preliminary article (see supplement material) [31]. The Cronbach’s alpha of scale for current study is good (0.875).

#### Dimensions of impulsivity

To measure the dimensions of impulsivity UPPS- Impulsive Behavior Scale [33] was used. The scale has 20 items and there are 4 items gauging each of the five dimensions of impulsivity (i.e., Negative Urgency, Lack of Perseverance, Lack of Premeditation, Sensation Seeking, Positive Urgency). Short UPPS-P has demonstrated a reliability of 0.64–0.70 and has been established as a widely used measure of impulsivity [34]. In the current study the reliability of the Short UPPS-P is 0.834.

#### Perceived stress

Perceived Stress Scale [35] was used to measure perceived stress. It has 10 items and is one of the most used measures of stress. Crosswell and Lockwood [20] stated that the reliability of PSS has been investigated in multiple studies across different populations and the reliability ranges from 0.77 to 0.82. so far it is the most well-constructed and thoroughly studied measure of perceived stress [20]. In the current study the reliability of the PSS is 0.794.

### Data analysis plan

The data were cleaned and analysed by using Statistical Package for Social Sciences (SPSS) version 26. The test performed included reliability analysis, descriptive statistics to explain the characteristics of sample and occurrence of pica and avoidant/restrictive food intake disorder, skewness and kurtosis to check the normality, Pearson correlation and Hayes’ PROCESS macro was used for mediation [36].

## Results

### Descriptive analysis

The mean subjective English proficiency rating of the respondents was 7.6 out of 10. The results shows that Mean Body Mass Index (BMI) of the participants was 21.19 (SD =  ± 2.2) (Table 1).

**Table 1:** Demographic characteristics of the sample (N = 660)

Characteristic	n (%)
*Gender*	
Male	221 (33.5%)
Female	427 (64.8%)
Other/prefer not specify	12 (1.7%)
*Year in university*	
First	41 (6.2%)
Second	273 (41.2%)
Third	180 (27.2%)
Fourth	150 (22.7%)
Fifth	15 (2.27%)
*Socioeconomic status*	
Upper class	34 (5.1%)
Upper middle class	270 (40.9%)
Middle class	306 (46.3%)
Lower middle class	26 (3.9%)
Lower class	6 (0.9%)
Prefer not specify	18 (2.7%)
*BMI category*	
Underweight	72 (10.9%)
Normal	498 (75.4%)
Overweight	67 (10.1%)
Obese	23 (3.45%)
*Ethnicity*	
Punjabi	211 (32.0%)
Urdu speaking	151 (22.9%)
Pashtoon	93 (14.1%)
Hazara	51 (7.7%)
Sindhi	35 (5.3%)
Gilgiti/Balti	32 (4.8%)
Baloch	22 (3.3%)
Memon	11 (1.6%)
Seraiki	12 (1.8%)
Kashmiri	4 (0.6%)
Other	4 (0.6)
Prefer not to specify	31 (4.7%)

### Research objective 1

The results suggest that twenty-eight (4.2%) participants reported most of the symptoms of pica and among them eleven pica on multiple items of scale. It was found that the frequency of the nonedible element consumed by the participants are range from 1 to 11. For instance, paper (6), clay (3), stone (1), tissue paper (1), aluminum foil wrap (1), chalk (1), raw rice (2), charcoal for sketching (1), sand (1), and multiple items (11). The list of multiple items consumed are mentioned in Table 2. Moreover, out of 28 people affected with pica only four reported that they want it treated as opposed to 17 individuals who clearly stated that they didn’t want treatment and seven who responded with a ‘maybe’ response. Only one respondent reported all of the symptoms of pica and ARFID listed on the questionnaire. 19 respondents (2.8%), comprising 13 females and six males fulfilled the criteria of avoidant/restrictive food intake disorder on the questionnaire. One observation was excluded because of scarcity of food in that area, as suggested by DSM-5. Different presentations of avoidant/restrictive food intake disorder were noted. 13 mixed presentations were reported with varying degrees of all three symptom categories namely sensory based food avoidance, lack of interest in food, and fear of negative consequences of eating. Three participants reported a combination of two symptom categories of sensory based food avoidance and lack of interest in food. Two participants reported a combination of lack of interest in food and fear of negative consequences. One participant reported avoidant/restrictive food intake disorder solely characterized by lack of interest in food. 17 out of 19 respondents reported that they were prescribed with some form of supplement to support nutrition and gain weight. None of the participants reported tube feeding.

**Table 2:** List of multiple item pica

Non-food items consumed by participants
Raw rice and clay
Chalk, sand, pencil, and paper
Plastic, paper, rubber, and gandhak
Pencil and raw rice
Slate, pencil, and clay
Paint chips and clay
Paper and pencil lead
Clay and chalk
Egg shell, paint, and chalk
Clay and cuttlebone
Cardboard and paper

### Research objective 2

The results affirm the significant positive relationship between presence of pica and each of the dimensions of impulsivity namely negative urgency, positive urgency, lack of premeditation, lack of perseverance, and sensation seeking at the significance level between 0.01 and 0.001. Also, presence of pica seems to be significantly positively associated with perceived stress i.e., r = 0.349 (*p* = 0.01). The bivariate correlations between the presence of avoidant/restrictive food intake disorder and each of the dimensions of impulsivity (see Table 3, 4) also revealed substantial positive associations at the significance level between 0.01 and 0.001. The relationship between avoidant/restrictive food intake disorder and perceived stress also had significant positive correlation i.e., r = 0.278 (*p* = 0.01).

**Table 3:** Correlations between pica and dimensions of impulsivity

Variable	Pica	NU	PU	LoPre	LoPer	SS	Stress
Pica	1						
NU	0.445**	1					
PU	0.377**	0.551**	1				
LoPre	0.389**	0.414**	0.433**	1			
LoPer	0.291**	0.332**	0.277**	0.520**	1		
SS	0.400**	0.502**	0.516**	0.405**	0.243**	1	
Stress	0.349**	0.399**	0.351**	0.267**	0.161**	0.357**	1

*NU* negative urgency, *PU* positive urgency, *LoPre* lack of premeditation, *LoPer* lack of perseverance, *SS* sensation seeking

**Correlation is significant at 0.01 level (one-tailed)

**Table 4:** Correlations between avoidant/restrictive food intake disorder, dimensions of impulsivity and perceived stress

Variable	ARFID	NU	PU	LoPre	LoPer	SS	Stress
ARFID	1						
NU	0.298**	1					
PU	0.272**	0.551**	1				
LoPre	0.296**	0.414**	0.433**	1			
LoPer	0.278**	0.332**	0.277**	0.520**	1		
SS	0.263**	0.502**	0.516**	0.405**	0.243**	1	
Stress	0.278**	0.399**	0.351**	0.267**	0.161**	0.357**	1

### Research objective 3

Five mediation models related to pica and each of the dimensions of impulsivity were thus tested using PROCESS macro. The Table 5, showed that the direct path from negative urgency and stress to pica is both significant and positive, means that higher degrees of negative urgency and stress are more likely to have pica than people who report lower levels of negative urgency. The indirect effect (value = 0.187) is also positive and significant and lies within the upper and lower bounds of the confidence interval thus affirming that perceived stress mediates the relationship between presence of pica and negative urgency (BootLLCI = 0.048, BootULCI = 0.421). However, the mediation analysis established perceived stress as a partial mediator because both direct and indirect effects are statistically significant, perceived stress accounts for 20.1% of variance in relationship between negative urgency and presence of pica. The direct path from positive urgency and stress to pica is both significant and positive, shows that higher degrees of positive urgency and stress are more likely to report pica than people who report lower levels of positive urgency. The indirect effect (value = 0.214) is also positive and significant and lies within the upper and lower bounds of the confidence interval thus shows that perceived stress partially mediates the relationship between presence of pica and positive urgency (BootLLCI = 0.108, BootULCI = 0.380). The perceived stress accounts for 29.9% of variance in the relationship between positive urgency and pica.

**Table 5:** Mediation analysis: total, direct, and indirect with pica as outcome variable and perceived stress as mediator (Preacher–Hayes bootstrap Test)

	Direct effects			Indirect effects			Total effects		
	Estimate	S.E	*p*	Estimate	S.E	*p*	Estimate	S.E	*p*
NU	0.755	0.107	0.001	0.187	0.063	0.000	0.944	0.145	0.000
PU	0.500	0.106	0.000	0.214	0.051	0.000	0.713	0.101	0.000
LoPre	0.620	0.109	0.000	0.177	0.067	0.000	0.686	0.111	0.000
LoPer	0.430	0.107	0.000	0.131	0.038	0.000	0.551	0.177	0.000
SS	0.510	0.095	0.000	0.208	0.050	0.000	0.828	0.121	0.000

NU negative urgency, PU positive urgency, LoPre lack of premeditation, LoPer lack of perseverance, SS sensation seeking

The direct path from lack of premeditation to pica is both significant and positive, which depicted that higher degrees of lack of premeditation are more likely to have pica than people who report lower levels of lack of premeditation. The direct effect of total perceived stress on pica is both positive and significant. The indirect effect (value = 0.177) is also positive and significant and lies within the upper and lower bounds of the confidence interval, thus affirming that perceived stress partially mediates the relationship between presence of pica and lack of premeditation (BootLLCI = 0.078, BootULCI = 0.322). The perceived stress only accounts for 9.7% of variance in relationship between lack of premeditation and pica. The path (direct effect) from of lack of perseverance to pica is both significant and positive, shows that higher degrees of lack of perseverance are more likely to have pica than people who report lower levels of negative urgency. The indirect effect (value = 0.131) is also positive and significant and lies within the upper and lower bounds of the confidence interval thus affirming that perceived stress mediates the relationship between presence of pica and lack of perseverance (BootLLCI = 11.913, BootULCI = 16.843). However, results shows that perceived stress act as a partial mediator because both direct and indirect effects are statistically significant, perceived stress accounts for 22% of variance in relationship between lack of perseverance and presence of pica. The direct path from sensation seeking and stress to pica is both significant and positive shows that higher degrees of sensation seeking and stress are more likely to have pica than people who report lower levels of sensation seeking. The indirect effect (value = 0.208) is also positive and significant and lies within the upper and lower bounds of the confidence interval thus affirming that perceived stress mediates the relationship between presence of pica and sensation seeking (BootLLCI = 0.105, BootULCI = 0.375). However, results illustrates that perceived stress as a partial mediator because both direct and indirect effects are statistically significant, perceived stress accounts for 38.5% of variance in the relationship between sensation seeking and presence of pica.

Table 6 shows that the direct path from negative urgency to avoidant/restrictive food intake disorder is both significant and positive. The direct effect of total perceived stress on avoidant/restrictive food intake disorder is both positive and significant. The indirect effect (value = 0.188) is also positive and significant and lies within the upper and lower bounds of the confidence interval thus affirming that perceived stress partially mediates the relationship between presence of avoidant/restrictive food intake disorder and negative urgency (BootLLCI = 0.027, BootULCI = 0.359). The perceived stress accounts for 32.7% of variance in relationship between negative urgency and presence of avoidant/restrictive food intake disorder. The path (direct effect) from positive urgency to avoidant/restrictive food intake disorder is both significant and positive. The direct effect of total perceived stress on avoidant/restrictive food intake disorder is both positive and significant. The indirect effect (value = 0.182) is also positive and significant and lies within the upper and lower bounds of the confidence interval thus affirming that perceived stress partially mediates the relationship between presence of avoidant/restrictive food intake disorder and positive urgency (BootLLCI = 0.066, BootULCI = 0.331). The perceived stress accounts for 35.6% of variance in relationship between negative urgency and presence of avoidant/restrictive food intake disorder.

**Table 6:** Mediation analysis: total, direct, and indirect with Avoidant/Restrictive Food Intake Disorderas outcome variable and perceived stress as mediator (Preacher–Hayes bootstrap test)

	Direct effects			Indirect effects			Total effects		
	Estimate	S.E	*p*	Estimate	S.E	*p*	Estimate	S.E	*p*
NU	0.385	0.112	0.001	0.188	0.067	0.000	0.573	0.145	0.000
PU	0.333	0.102	0.000	0.184	0.047	0.000	0.517	0.101	0.000
LoPre	0.378	0.105	0.000	0.147	0.043	0.000	0.525	0.111	0.000
LoPer	0.478	0.106	0.000	0.100	0.032	0.000	0.578	0.177	0.000
SS	0.338	0.047	0.000	0.173	0.047	0.000	0.511	0.099	0.000

NU negative urgency, PU positive urgency, LoPre lack of premeditation, LoPer lack of perseverance, SS sensation seeking

The direct path from lack of premeditation to avoidant/restrictive food intake disorder is both significant and positive. The direct effect of total perceived stress on avoidant/restrictive food intake disorder is both positive and significant. The indirect effect (value = 0.147) is also positive and significant and lies within the upper and lower bounds of the confidence interval thus affirming that perceived stress partially mediates the relationship between presence of avoidant/restrictive food intake disorder and lack of premeditation (BootLLCI = 0.048, BootULCI = 0.278). The perceived stress accounts for 28% of variance in relationship between lack of premeditation and presence of avoidant/restrictive food intake disorder. The direct path from negative urgency to avoidant/restrictive food intake disorder is both significant and positive. The direct effect of total perceived stress on avoidant/restrictive food intake disorder is both positive and significant. The indirect effect (value = 0.100) is also positive and significant and lies within the upper and lower bounds of the confidence interval thus affirming that perceived stress mediates the relationship between presence of avoidant/restrictive food intake disorder and lack of perseverance (BootLLCI = 0.029, BootULCI = 0.197). Perceived stress doesn’t not mediate the relationship between lack of perseverance and presence of avoidant/restrictive food intake disorder since it only accounts for 17.4% variance.

The direct path from sensation seeking to avoidant/restrictive food intake disorder is both significant and positive. The direct effect of total perceived stress on avoidant/restrictive food intake disorder is both positive and significant. The indirect effect (value = 0.173) is also positive and significant and lies within the upper and lower bounds of the confidence interval thus affirming that perceived stress partially mediates the relationship between presence of avoidant/restrictive food intake disorder and sensation seeking (BootLLCI = 0.058, BootULCI = 0.314). The perceived stress accounts for 33.9% of variance in relationship between sensation seeking and presence of avoidant/restrictive food intake disorder.

## Discussion

The current study indicates towards the probability of presence of both pica and avoidant/restrictive food intake disorder among young adults in Pakistan. Since the percentage of people affected by both pica and avoidant/restrictive food intake disorder seems to be comparable to the global statistics, there’s an increased likelihood of finding similar prevalence rates if a wider epidemiological investigation is carried out at national level. In the current study, 4.2% of the participants met all the criteria for pica, which compliments the global prevalence rates which range from 0.3 to 4% [37]. The rates are also comparable to the neighbouring countries of Pakistan such as Iran [38] and India [39]. Pica was not found to be associated with any cultural, religious, or social practice for this sample. The findings contrast with those from India, where pica can be a part of religious rituals and cultural practices [39], it is also interesting since Pakistan shares a common cultural heritage with India. In addition, 60% of the participants reported that they have been consuming inedible items from five years or less, considering that only young adults (18–24 years were included in this study for them the onset of pica would have been in their teen years. The trends of onset of pica are understudied too [40] and the current study has elicited a need to study pica in groups other than children and pregnant women, and in different age groups which may help researchers investigate factors related to onset further.

2.8% of the participants in this study met full criteria for avoidant/restrictive food intake disorder, a frequency which is comparable to that reported in the literature across the world [41]. Four different presentations of avoidant/restrictive food intake disorder were identified in the current sample, the mixed presentation was predominant and most of the individuals with avoidant/restrictive food intake disorder exhibited mixed symptomology. Five individuals reported an overlap of two symptom clusters, and one individual reported avoidant/restrictive food intake disorder marked by presence of one symptom clusters only. It affirmed that avoidant/restrictive food intake disorder is a heterogeneous disorder and often individuals report symptoms of more than one subtype [42]. Thus, assuming that there are only three subtypes, and a mixed presentation type can be too simplistic. It appears that restrictive eating behavior can have different presentations with distinct etiologies, and symptom clusters can overlap without being mutually exclusive as suggested by Thomas et al. [43]. Clinicians should therefore assess the restrictive eating behavior and its patterns carefully so that they can effectively manage each of the unique presentation of avoidant/restrictive food intake disorder they come across with. Moreover, the results are consistent with the literature that eating problems are more common among females as compared to males [9, 44]. In Pakistani culture females have developed eating problems due to malnutrition, stress or public pressure to remain smart and look beautiful [45].

The relationship between each of the dimensions of impulsivity and pica was found to be true, the relationship between pica and negative urgency was strongest. The results of the current study align with the findings of Gustavson et al. [46], who reported similar genetic correlations between each of the dimensions of impulsivity and eating disorders. The approach was different, yet the results are the same, which highlights the need for further exploration of the link between dimensions of impulsivity and pica. The findings affirm that unhealthy eating behaviors appear to be linked to an emphasis on meeting one's immediate need or acting on an instant impulse without adequate consideration of their behavior's potential negative implications in terms of one's long-term interests, aspirations, or health [47], and such might be applicable to pica behavior as well. People with positive and negative urgency find it hard to process control their emotions and they engage in impulsive behaviors related to eating in order to feel better [33]. The role of lack of premeditation and lack of perseverance can be understood if it is considered that people who engage in such unhealthy eating behaviors discount the negative effects of such behaviors in long term [13]. Individuals with eating disorders which are marked by oral sensory overstimulation (e.g., bulimia nervosa) are more likely to score higher on sensation seeking compared to those with eating disorders involving restrictive eating behaviors [17]. In pica, the need for oral sensory stimulation is a central theme, the chewing and mouthing of the non-food item bring pleasure [48]. The findings of current study also reported the relationship between pica and avoidant/restrictive food intake disorder with sensation seeking. Perceived stress was also established as mediator between four of the dimensions of impulsivity (Negative Urgency, Positive Urgency, Lack of Perseverance, and Sensation Seeking and pica. Besides, perceived stress partial mediated the relationship between dimensions of impulsivity and the presence of avoidant/restrictive food intake disorder. However, partial mediation was established instead of a complete one, which highlighted that there are some other factors as well which play a role, which need to be explored in future research. Partial mediation is understandable since perceived stress alone cannot account for pica behavior since in psychological processes there are lots of other personality related factors, situational factors, temporal factors, and sociodemographic factors which might intervene as well. Also, a single interaction between a personality variable and stress cannot explain the underlying psychological process related to an eating disorder in entirety [49]; although it may indicate that interaction of certain factors is of significance and might entail a risk for instance, socio-economic status, social influences, other personality traits, comorbid mental health condition, and body image etc. [50].

The literature on pica and avoidant/restrictive food intake disorder is limited, and research on both of these disorders is in its preliminary phases. The current study had a unique standing in both local and global context. Its global significance is associated with the fact that there is limited research available on both of these disorders and particularly involving young adults. In the local context, it was the first study which was designed to study pica and avoidant/restrictive food intake disorder among Pakistani young adults. The current research has established that there is presence of pica and avoidant/restrictive food intake disorder among Pakistani young adults, these disorders may go unnoticed or are underreported but are not non-existent. It was established that there is much more to be explored and investigated about these disorders in the context of Pakistan; thus, future research can utilize more rigorous research methods and larger epidemiological surveys to determine exact prevalence rates and develop a detailed understanding of these eating disorders. A major drawback of this study is that the generalizability of this study is limited due to the use of non-probability sampling technique. Also, the study was restricted to a certain age group i.e., young adults, which made it specific. In addition to that, multiple respondent format was not used so the screening of the disorders was entirely dependent on the self-report so there might be some issues with accuracy.

## Conclusion

In conclusion, the current study was the preliminary study on pica and avoidant/restrictive food intake disorder in Pakistani context, it has confirmed the presence of pica and avoidant/restrictive food intake disorder in a specific stratum (i.e., university going young adults) of Pakistan and elicited the need to further investigate these disorders among other age groups and with more precise methods. In addition to this, the findings highlight the need to include impulsivity and stress while developing intervention and prevention program for the youngster who may report symptoms of pica and avoidant/restrictive food intake disorder.

## Data Availability

The datasets used in this study are not publicly available because confidentially of the participants are compromised and the legal rights related to data, impede participant’s ethical issues of anonymity.
